# Association of *NCOA3* polymorphisms with Dyslipidemia in the Chinese Han population

**DOI:** 10.1186/s12944-015-0126-y

**Published:** 2015-10-09

**Authors:** Mingxi Yu, Siame Gilbert, Yong Li, Huiping Zhang, Yichun Qiao, Yuping Lu, Yuan Tang, Qing Zhen, Yi Cheng, Yawen Liu

**Affiliations:** Department of Epidemiology and Biostatistics, School of Public Health, Jilin University, Changchun, 130021 China; Department of Psychiatry, Yale University School of Medicine, VA Medical, Center/116A2, 950 Campbell Avenue, West Haven, CT 06516 USA; The Cardiovascular Center, The First Hospital of Jilin University, Changchun, 130021 China

**Keywords:** NCOA3, Polymorphism, Dyslipidemia

## Abstract

**Background:**

Nuclear receptor coactivator-3 (NCOA3) is involved in various physiological processes. Emerging evidence from previous studies using animal models suggests that the NCOA3 gene (*NCOA3*) plays a critical role in lipid metabolism as well as adipogenesis and obesity. The present study aims to investigate the association between *NCOA3* SNPs and dyslipidemia in the Chinese Han population.

**Methods:**

Five hundred and twenty-nine (529) Chinese Han subjects were recruited. Four tag SNPs (rs2425955G > T, rs6066394T > C, rs10485463C > G, and rs6094753G > A) in *NCOA3*, selected from the HapMap website, were genotyped using MALDI-TOF mass spectrometry. Data analysis was performed using SPSS 16.0, SNPStats and haploview 4.2.

**Results:**

Four SNPs (rs2425955, rs6066394, rs10485463, and rs6094753) were associated with triglyceride levels. Except for SNP rs10485463, genotype distributions and allele frequencies of the other three NCOA3 SNPs (rs2425955, rs6066394, and rs6094753) were significantly different between hypertriglyceridemia subjects and normal group. Significant differences were also observed in allele frequencies and genotype distributions of SNP rs10485463 between low-HDL cholesterolemia subjects and normal group. Carriers of rs2425955 T allele had a lower risk of hypertriglyceridemia compared to GG genotype. Similar results were observed from rs6094753. Subjects with rs6066394 CT genotype had a lower risk of hypertriglyceridemia than those with the TT genotype; however, CC and TT genotypes showed no significant difference in the risk of hypertriglyceridemia. Similar results were found in the association between rs6066394 and hypercholesterolemia. The variant alleles of rs2425955, rs6066394 and rs6094753 were associated with a lower risk of hypertriglyceridemia compared with the wild-type alleles. The G allele of rs10485463 was associated with an increased risk of low-HDL cholesterolemia. In the log-additive model the association between rs2425955 and hypertriglyceridemia remained significant after Bonferroni correction, and genotypes with variant alleles were associated with a lower risk of hypertriglyceridemia.

**Conclusions:**

In summary, this study demonstrated that variation in *NCOA3* might influence the risk of dyslipidemia and serum lipid levels in Chinese Han population.

## Background

Nuclear receptor coactivator-3 (*NCOA3*), a member of the p160 family of nuclear receptor coactivators, plays significant roles in various physiological processes, such as mammary gland development, cell reproduction, somatic growth, and female generative function [1]. NCOA3 gene, consisting of 22 exons, is located in chromosomal region 20q12 and its mRNA was expressed in various tissues, such as muscle, heart, liver, brain, mammary gland [2–7].

Previous studies provided evidence that the NCOA3 gene (*NCOA3*) plays a primary role in adipogenesis as well as obesity by regulating the gene expression of peroxisome proliferator-activated receptor γ (PPARγ) - the master regulator of adipocyte development and differentiation [8–16]. These studies demonstrated that NCOA3 could enhance the transcriptional activity of PPARγ which promotes obesity, and that adipocyte differentiation was suppressed in *NCOA3* knockout mice with a significantly decreased gene expression of PPARγ [8–11]. Besides, the researchers found that NCOA3 deficient mice redistributed fat between visceral adipose tissue (VAT) and subcutaneous adipose tissue (SCAT) under high-fat diet (HFD), and that *NCOA3* ablation led to a significantly higher VAT/SCAT weight ratio under HFD versus normal diet - providing an evidence of the role in redistributing fat distribution for NCOA3 [17]. Studies also revealed that obese people with VAT fat accumulation were more tightly related to the risk of suffering from diabetes mellitus and dyslipidemia than those with a normal fat distribution [18, 19].

In addition to its crucial role in controlling adipogenesis and fat distribution, emerging evidence from metabolic studies [20–22] has shown that NCOA3 can participated in metabolic control and energy homeostasis. Ma et al. [20] reported that deletion of NCOA3 ameliorated hepatic steatosis and lipid accumulation in mice fed with a high-fat diet. Thus, NCOA3 plays a critical role in regulating hepatic lipid metabolism. A study by Coste et al. [22] demonstrated that caloric excess induced *NCOA3* expression, leading to the restraint of activity of PPARγ coactivator-1α (PGC-1α, which is the coordinator of mitochondrial function) and decreased energy expenditure (EE), while caloric restriction reduced NCOA3 levels, resulting in improved PGC-1α activity, increased EE and an improved metabolic profile, such as lower fasting cholesterol, triglycerides, and free fatty acids levels. Disturbance in energy homeostasis, which is sustained by a balance between energy intake and energy expenditure, may lead to metabolic diseases such as obesity, dyslipidemia, and atherosclerosis [23].

Dyslipidemia is a significant risk factors for coronary heart disease, which is a major public health problem in the worldwide. Although the exact cause of dyslipidemia is unknown, blood lipid level is influenced by multiple genetic and environmental factors and their interactions [24, 25]. It is clear from the published literature that *NCOA3* plays a critical role in adipogenesis, energy homeostasis, and lipid metabolism. However, to the best of our knowledge, studies on NCOA3 gene so far are carried out mainly on animal models, with no published epidemiologic studies that investigated the association of human *NCOA3* single nucleotide polymorphisms (SNPs) and metabolic disorders. In this study, we intended to analyze the association between polymorphisms of human *NCOA3* and dyslipidemia.

## Results

47.6 % of the respondents were males, with an average age of 58 ± 10 years. The percentages of subjects with hypertriglyceridemia, hypercholesterolemia, low-HDL cholesterolemia, and hyper-LDL cholesterolemia were 30.6, 18.7, 15.9, and 30.1 %, respectively. Table 1 shows the average plasma level of lipids.

**Table 1:** Characteristics of study subjects

Characteristics	Men	Women	Total
	(*n* = 252)	(*n* = 277)	(*n* = 529)
Age, years	58 ± 10	60 ± 9	59 ± 10
Hypertriglyceridemia, *n* (%)	85(33.7)	77(27.8)	162(30.6)
Hypercholesterolemia, *n* (%)	38(15.1)	61(22.0)	99(18.7)
High LDL, *n* (%)	30(11.9)	54(19.5)	84(15.9)
Reduced HDL, *n* (%)	101(40.1)	58(20.9)	159(30.1)
TG, mmol/l	2.28 ± 2.36	2.07 ± 1.91	2.17 ± 2.14
TC, mmol/l	5.08 ± 1.06	5.32 ± 1.18	5.21 ± 1.13
LDL, mmol/l	3.02 ± 0.99	3.28 ± 1.03	3.16 ± 1.02
HDL, mmol/l	1.23 ± 0.44	1.35 ± 0.42	1.30 ± 0.43

Means ± standard deviation for age, TG, TC, LDL, and HDL

The four SNPs were significantly associated with plasma levels of triglyceride (*P* = 0.009, 0.004, 0.019, and 0.006, respectively), and subjects carrying the variant allele of the four SNPs had a lower level of triglyceride than those who were homozygous for the wild-type allele. However, the significant association between these four SNPs and three other serum lipid parameters (TC, LDL and HDL) were not observed (Table 2).

**Table 2:** The association between *NCOA3* polymorphisms and serum lipid

SNP	Genotype	TC(mmol/l)	LDL(mmol/l)	TG(mmol/l)	HDL(mmol/l)
rs10485463	CC(*n* = 247)	5.2 ± 1.2	3.1 ± 0.9	2.3 ± 2.2	1.3 ± 0.4
	CG(*n* = 193)	5.1 ± 1.2	3.1 ± 1.0	2.1 ± 2.2	1.3 ± 0.4
	GG(*n* = 40)	5.1 ± 1.1	3.2 ± 0.9	1.6 ± 0.9	1.2 ± 0.4
P		0.669	0.795	0.009	0.056
rs2425955	GG(*n* = 269)	5.4 ± 1.1	3.2 ± 1.1	2.4 ± 3.1	1.3 ± 0.4
	GT(*n* = 206)	5.1 ± 1.1	3.1 ± 0.9	1.9 ± 1.8	1.3 ± 0.4
	TT(*n* = 36)	4.9 ± 1.0	3.0 ± 0.8	1.5 ± 0.8	1.3 ± 0.4
P		0.051	0.707	0.004	0.801
rs6066394	CC(*n* = 65)	5.1 ± 1.3	3.1 ± 0.8	2.5 ± 3.0	1.3 ± 0.3
	CT(*n* = 223)	5.1 ± 1.0	3.1 ± 0.9	2.1 ± 2.5	1.3 ± 0.4
	TT(*n* = 217)	5.3 ± 1.2	3.2 ± 1.1	1.9 ± 1.8	1.3 ± 0.4
P		0.070	0.590	0.019	0.581
rs6094753	GG(*n* = 283)	5.3 ± 1.1	3.2 ± 1.1	2.9 ± 3.8	1.3 ± 0.4
	GA(*n* = 201)	5.1 ± 1.1	3.1 ± 0.9	2.4 ± 2.4	1.3 ± 0.4
	AA(*n* = 35)	5.0 ± 1.1	3.2 ± 0.9	1.9 ± 1.7	1.2 ± 0.4
P		0.101	0.682	0.006	0.444

Data was represented as mean ± SD, and *P* value was calculated with the general linear model adjusted for age and sex

The genotype distributions of the four *NCOA3* SNPs in the normal group conformed to Hardy–Weinberg equilibrium (HWE) (data not shown). Allele frequencies and genotype distributions of the four SNPs in *NCOA3* are presented in Table 3. Except for SNP rs10485463, genotype distributions and allele frequencies of the other three *NCOA3* SNPs (rs2425955, rs6066394, and rs6094753) were significantly different between hypertriglyceridemia subjects and normal group. Significant differences were also observed in allele frequencies and genotype distributions of SNP rs10485463 between low-HDL cholesterolemia subjects and normal group.

**Table 3:** Genotype distributions and allele frequencies in dyslipidemia and normal groups

SNPs	Hypercholesterolemia	Normal group	Hypertriglyceridemia	Normal group	High LDL	Normal group	Reduced HDL	Normal group
rs10485463								
CC	48(53.9)	199(50.9)	87(57.6)	160(48.6)	35(49.3)	212(51.8)	61(44.5)	186(54.2)
CG	33(37.1)	160(40.9)	54(35.8)	139(42.2)	30(42.3)	163(39.9)	58(42.3)	135(39.4)
GG	8(9.0)	32(8.2)	10(6.6)	30(9.1)	6(8.5)	34(8.3)	18(13.1)	22(6.4)
*χ* ^*2*^	0.452		3.48		0.164		7.319	
P	0.798		0.175		0.921		0.026	
C	129(72.5)	558(71.4)	228(75.5)	459(69.8)	100(70.4)	587(71.8)	180(65.7)	507(73.9)
G	49(27.5)	224(28.6)	74(24.5)	199(30.2)	42(29.6)	231(28.2)	94(34.3)	179(26.1)
*χ* ^*2*^	0.089		3.351		0.106		6.49	
P	0.766		0.067		0.744		0.011	
rs2425955								
GG	57(60.0)	212(51.0)	99(62.3)	170(48.3)	42(52.5)	227(52.7)	81(54.4)	188(51.9)
GT	34(35.8)	172(41.3)	54(34.0)	152(43.2)	35(43.8)	171(39.7)	58(38.9)	148(40.9)
TT	4(4.2)	32(7.7)	6(3.8)	30(8.5)	3(3.8)	33(7.7)	10(6.7)	26(7.2)
*χ* ^*2*^	3.123		9.876		1.740		0.252	
P	0.210		0.007		0.419		0.882	
G	148(77.9)	596(71.6)	252(79.2)	492(69.9)	119(74.4)	625(72.5)	220(73.8)	534(72.4)
T	42(22.1)	236(28.4)	66(20.8)	212(30.1)	41(25.6)	237(27.5)	78(26.2)	200(27.6)
*χ* ^*2*^	3.061		9.689		0.238		0.224	
P	0.080		0.002		0.626		0.636	
rs6066394								
TT	49(52.7)	168(40.8)	81(51.9)	138(39.0)	36(45.6)	181(42.5)	67(45.6)	150(41.9)
CT	33(35.5)	190(46.1)	56(35.9)	167(47.9)	35(44.3)	188(44.1)	63(42.9)	160(44.7)
CC	11(11.8)	54(13.1)	19(12.2)	46(13.2)	8(10.1)	57(13.4)	17(11.6)	48(13.4)
*χ* ^*2*^	4.554		7.783		0.696		0.683	
P	0.103		0.020		0.706		0.711	
T	131(70.4)	526(63.8)	218(69.9)	443(63.1)	107(67.7)	550(64.6)	197(67.0)	460(64.2)
C	55(29.6)	298(36.2)	94(30.1)	259(36.9)	51(32.3)	302(35.4)	97(33.0)	256(35.8)
*χ* ^*2*^	2.903		4.618		0.588		0.699	
P	0.088		0.032		0.443		0.403	
rs6094753								
GG	60(61.9)	223(52.8)	101(63.5)	182(50.6)	44(53.7)	239(54.7)	86(55.5)	197(54.1)
GA	30(30.9)	171(40.5)	52(32.7)	149(41.4)	32(39.0)	169(38.7)	56(36.1)	145(39.8)
AA	7(7.2)	28(6.6)	6(3.8)	29(8.1)	6(7.3)	29(6.6)	13(8.4)	22(6.0)
*χ* ^*2*^	3.089		8.547		0.063		1.308	
P	0.213		0.014		0.969		0.520	
G	150(77.3)	617(73.1)	254(79.9)	513(71.2)	120(73.2)	647(74.0)	228(73.5)	539(74.0)
A	44(22.7)	227(26.9)	64(20.1)	207(28.8)	44(26.8)	227(26.0)	82(26.5)	189(26.0)
*χ* ^*2*^	1.453		8.504		0.053		0.027	
P	0.228		0.004		0.819		0.869	

Logistic regression analysis revealed that hypertriglyceridemia was associated with three *NCOA3* SNPs (rs2425955, rs6066394, and rs6094753). Subjects carrying the variant allele of SNP rs2425955 (genotypes: GT and TT) had a lower risk of developing hypertriglyceridemia compared to those who were homozygous for the wild-type allele G (GT vs. GG: OR = 0.60, 95 % CI = 0.40–0.89; TT vs. GG: OR = 0.34, 95 % CI = 0.14–0.86). Similar results were obtained from SNP rs6094753 (GA vs. GG: OR = 0.62, 95 % CI = 0.41–0.92; AA vs. GG: OR = 0.37, 95 % CI = 0.15–0.94). Subjects with the CT genotype of rs6066394 had a lower risk of hypertriglyceridemia than those with the TT genotype (OR = 0.55, 95 % CI = 0.37–0.83); but those with the CC and TT genotypes showed no significant differences in the risk of suffering from hypertriglyceridemia (OR = 0.70, 95 % CI = 0.38–1.27). Besides, the CT genotype of rs6066394 was slightly associated with a lower risk of hypercholesterolemia compared to the TT genotype (OR = 0.61, 95 % CI = 0.37–1.00). Similarly, the CC and TT genotypes of rs6066394 showed no significant differences in the risk of developing hypercholesterolemia (OR = 0.70, 95 % CI = 0.34–1.44). The GG genotype of SNP rs10485463 was associated with low-HDL cholesterolemia in comparison to the CC genotype (OR = 2.27, 95 % CI = 1.12–4.61); however, there was no significant difference between the CG and CC genotypes in affecting HDL levels (OR = 1.28, 95 % CI = 0.83–1.97).

In addition, we explored the association of the allele of the four SNPs with dyslipidemia. As shown in Table 4, the variant alleles of rs2425955 and rs6094753 were associated with a lower risk of hypertriglyceridemia compared with the wild-type alleles of these two SNPs (rs2425955: OR = 0.61, 95 % CI = 0.44–0.83; rs6094753: OR = 0.62, 95 % CI = 0.45–0.86). The C allele of SNP rs6066394 was significantly associated with a lower risk of hypertriglyceridemia (OR = 0.73, 95 % CI = 0.54–0.97). Compared with the C allele of SNP rs10485463, the G allele of SNP rs10485463 was associated with low-HDL cholesterolemia (OR = 1.48, 95 % CI = 1.09–2.00).

**Table 4:** Odds ratios for the likelihood of dyslipidemia according to *NCOA3* polymorphisms

SNPs	Hypercholesterolemia		Hypertriglyceridemia		High LDL		Reduced HDL	
	OR(95 % CI)	OR^a^(95 % CI)	OR(95 % CI)	OR^a^(95 % CI)	OR(95 % CI)	OR^a^(95 % CI)	OR(95 % CI)	OR^a^(95 % CI)
rs10485463								
CC	1.00	1.00	1.00	1.00	1.00	1.00	1.00	1.00
CG	0.85(0.52–1.39)	0.87(0.53–1.42)	0.71(0.47–1.07)	0.70(0.47–1.06)	1.11(0.66–1.89)	1.15(0.67–1.96)	1.31(0.86–2.00)	1.28(0.83–1.97)
GG	1.04(0.45–2.39)	1.09(0.47–2.54)	0.61(0.29–1.31)	0.57(0.26–1.23)	1.07(0.42–2.73)	1.19(0.46–3.07)	2.49(1.25–4.96)	2.27(1.12–4.61)
C	1.00		1.00		1.00		1.00	
G	0.95(0.66–1.36)		0.75(0.55–1.02)		1.07(0.72–1.58)		1.48(1.09–2.00)	
rs2425955								
GG	1.00	1.00	1.00	1.00	1.00	1.00	1.00	1.00
GT	0.73(0.46–1.18)	0.75(0.47–1.20)	0.61(0.41–0.91)	0.60(0.40–0.89)	1.11(0.68–1.81)	1.14(0.70–1.87)	0.91(0.61–1.36)	0.87(0.57–1.31)
TT	0.46(0.16–1.37)	0.46(0.16–1.36)	0.34(0.14–0.85)	0.34(0.14–0.86)	0.49(0.14–1.68)	0.49(0.14–1.67)	0.89(0.41–1.94)	0.89(0.40–1.97)
G	1.00		1.00		1.00		1.00	
T	0.72(0.49–1.04)		0.61(0.44–0.83)		0.91(0.62–1.33)		0.93(0.68–1.26)	
rs6066394								
TT	1.00	1.00	1.00	1.00	1.00	1.00	1.00	1.00
CT	0.59(0.37–0.97)	0.61(0.37–1.00)	0.56(0.37–0.85)	0.55(0.37–0.83)	0.94(0.56–1.56)	0.97(0.58–1.62)	0.88(0.58–1.33)	0.83(0.54–1.26)
CC	0.70(0.34–1.44)	0.70(0.34–1.44)	0.69(0.38–1.26)	0.70(0.38–1.27)	0.71(0.31–1.60)	0.70(0.31–1.60)	0.79(0.42–1.48)	0.79(0.42–1.50)
T	1.00		1.00		1.00		1.00	
C	0.74(0.52–1.05)		0.73(0.54–0.97)		0.87(0.60–1.25)		0.88(0.66–1.18)	
rs6094753								
GG	1.00	1.00	1.00	1.00	1.00	1.00	1.00	1.00
GA	0.65(0.40–1.05)	0.66(0.41–1.07)	0.63(0.42–0.94)	0.62(0.41–0.92)	1.03(0.63–1.69)	1.06(0.64–1.74)	0.88(0.59–1.32)	0.84(0.56–1.26)
AA	0.93(0.39–2.23)	0.92(0.38–2.21)	0.37(0.15–0.93)	0.37(0.15–0.94)	1.12(0.44–2.86)	1.11(0.43–2.85)	1.35(0.65–2.81)	1.42(0.67–3.00)
G	1.00		1.00		1.00		1.00	
A	0.80(0.55–1.15)		0.62(0.45–0.86)		1.04(0.72–1.52)		1.03(0.76–1.39)	

^a^ORs and 95 % CI were adjusted for age and sex

The association of *NCOA3* polymorphisms and dyslipidemia was further investigated through inheritance models. Only rs2425955 was significantly associated with hypertriglyceridemia after Bonferroni correction (*P* = 0.0010) (Table 5), and subjects with the variant allele had a lower risk of developing hypertriglyceridemia (OR = 0.59, 95 % CI = 0.43–0.82). However, no significant association between the four SNPs and dyslipidemia (hypercholesterolemia, low-HDL cholesterolemia, and hyper-LDL cholesterolemia) was observed in any inheritance models (data not shown).

**Table 5:** The association of *NCOA3* SNPs with Hypertriglyceridemia risk in inheritance models

SNPs	Model	Genotype	OR^a^(95 % CI)	*P*
rs10485463	Codominant	CC	1.00	0.1300
		CG	0.70(0.47–1.06)	
		GG	0.57(0.26–1.23)	
	Dominant	CC	1.00	0.0500
		CG + GG	0.68(0.46–1.00)	
	Recessive	CC + CG	1.00	0.2700
		GG	0.66(0.31–1.40)	
	Overdominant	CC + GG	1.00	0.0450
		CG	0.76(0.51–1.13)	
	log-additive		0.73(0.53–1.00)	
rs2425955	Codominant	GG	1.00	0.0048
		GT	0.60(0.40–0.89)	
		TT	0.34(0.14–0.85)	
	Dominant	GG	1.00	0.0025
		GT + TT	0.56(0.38–0.82)	
	Recessive	GG + GT	1.00	0.0400
		TT	0.42(0.17–1.03)	
	Overdominant	GG + TT	1.00	0.0390
		GT	0.66(0.45–0.98)	
	log-additive		0.59(0.43–0.82)	0.0010
rs6066394	Codominant	TT	1.00	0.0170
		TC	0.55(0.37–0.83)	
		CC	0.70(0.38–1.27)	
	Dominant	TT	1.00	0.0057
		TC + CC	0.58(0.40–0.86)	
	Recessive	TT + TC	1.00	0.7900
		CC	0.93(0.52–1.64)	
	Overdominant	TT + CC	1.00	0.0094
		TC	0.60(0.41–0.89)	
	log-additive		0.73(0.55–0.98)	0.0310
rs6094753	Codominant	GG	1.00	0.0100
		GA	0.62(0.41–0.92)	
		AA	0.37(0.15–0.93)	
	Dominant	GG	1.00	0.0048
		GA + AA	0.58(0.39–0.85)	
	Recessive	GG + GA	1.00	0.0620
		AA	0.45(0.18–1.11)	
	Overdominant	GG + AA	1.00	0.0480
		GA	0.68(0.46–1.00)	
	log-additive		0.61(0.44–0.85)	0.0025

^a^The ORs and 95 % CI were adjusted for age and gender. *P* < 0.0025 was considered statistically significant due to bonferroni corrected *P*-value = 0.0025

**Fig. 1:**
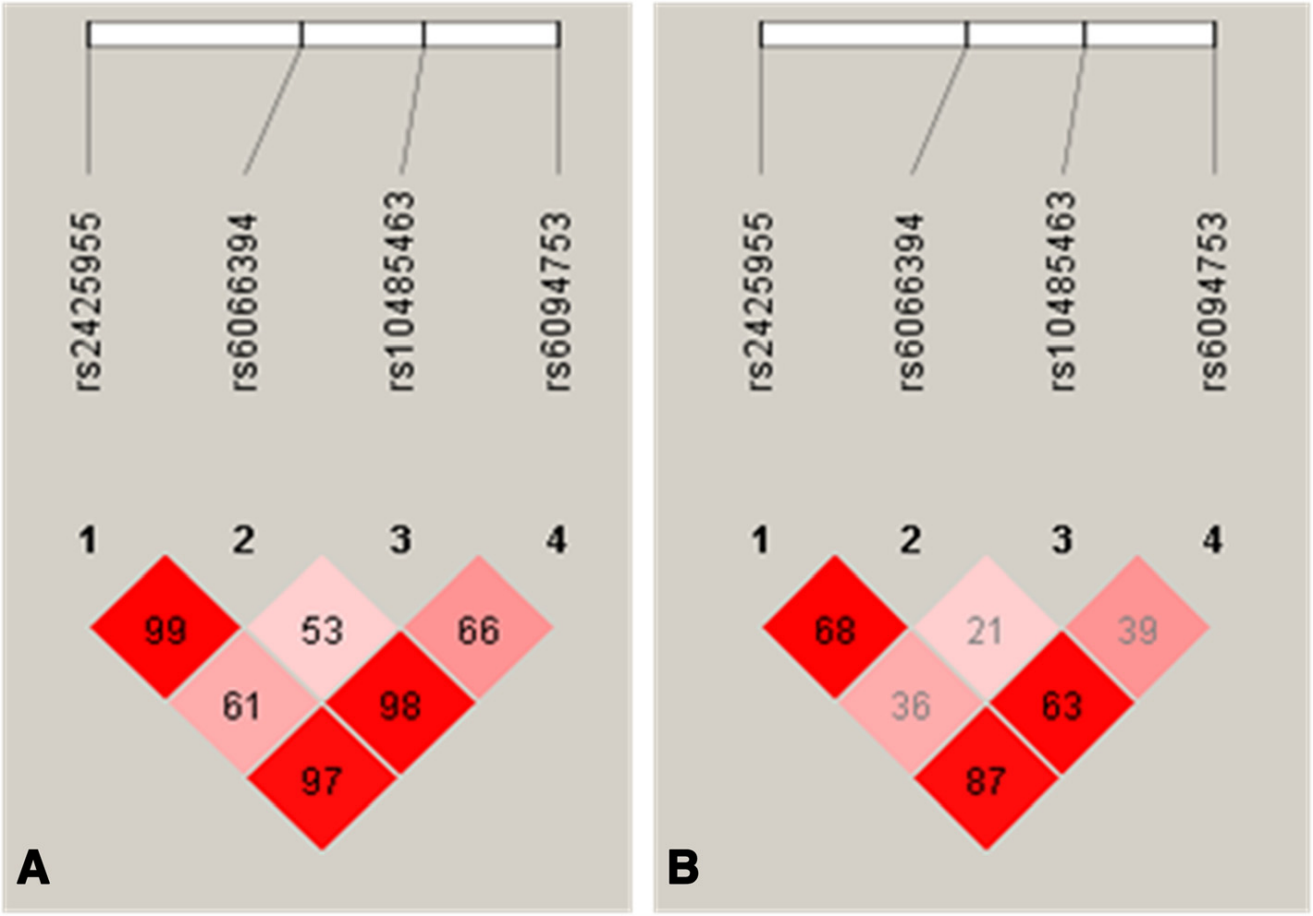
Linkage disequilibrium structure and relative chromosomal positions of the four SNPs. **a** Linkage disequilibrium plot showing D’ multiplying 100; (**b**) Linkage disequilibrium plot showing r^2^ multiplying 100

Figure 1 displays the linkage disequilibrium (LD) of the 4 SNPs in *NCOA3*. Except rs10485463, three other SNPs were in close LD (D’ ≥ 0.97, *r*^*2*^ ≥ 0.63).

## Discussion

In this study, our findings indicated that variation in *NCOA3* might influence the risk of dyslipidemia and serum lipid levels in Chinese Han population.

Previous studies have now convincingly demonstrated that the NCOA3 gene plays a critical role in adipogenesis by controlling the expression of PPARγ gene which is the master regulator of adipocyte development and differentiation [8–16]. In animal models, *NCOA3* ablation leads to reduced body weight and adipocyte volume due to impaired white adipocyte differentiation, and a significant decrease in the expression of PPARγ, while re-expression of *NCOA3* restored the adipocyte differentiation program. During adipocyte differentiation, NCOA3 served as coactivator that synergistically facilitates the gene expression of PPARγ with specific transcriptional factors of the CCAAT/enhancer-binding proteins(C/EBP) [8, 9].

PPARγ, a member of the nuclear hormone receptors that can interacts with its binding partner - retinoid X receptor α, can bind to DNA within the promoter elements of target genes [26]. PPARγ, predominantly found in adipose tissue and macrophages, functions as a regulator for adipocyte differentiation, lipid storage, glucose metabolism and play a vital role in the transcriptional regulation of a number of genes involved in metabolism, such as endothelial lipoprotein lipase (LPL), fatty acid translocase (FAT) and uncoupling protein (UCP) [26, 27]. In the regulation of lipid metabolism, PPARγ enhances plasmatic TG-rich liproprotein hydrolysis through directly regulating the expression of LPL and promotes uptake of fatty acid into adipocytes by controlling the expression of FAT, also called CD36 in macrophages [28, 29]. Hence, PPARγ promotes fat storage and reduces plasma lipid levels, especially plasma levels of triglyceride.

Hence, the diverse biological functions of PPARγ, such as regulation in lipid metabolism, lipid storage and so on, might be influenced by the NCOA3 gene through controlling PPARγ gene expression. And it can be further concluded that the NCOA3 gene might have association with plasma lipid levels and dyslipidemia, especially with hypertriglyceridemia.

In the present study, our data showed that four* NCOA3* SNPs were associated with plasma levels of triglyceride, and except rs10485436, three other SNPs of NCOA3 gene were associated with a decreased risk of hypertriglyceridemia. In the further inheritance model analysis, only rs2425955 was significantly associated with hypertriglyceridemia after Bonferroni correction.

SNP rs2425955 is in linkage disequilibrium (LD) with rs2076546 (D’ = 1) based on the Chinese HapMap data. SNP rs2076546 is in the coding region of NCOA3 gene. Although rs2076546 is a synonymous SNP and causes no change from threonine [30], the study of Burwinkel et al. [31] showed that polymorphism rs2076546 provoked a shift to less preferred codon ACG from the more preferred codon ACA in NCOA3 gene. The altered codon usage may decrease NCOA3 gene transcriptional and/or translational level [31–34]. Previous studies reported that NCOA3 facilitates PPARγ gene expression [8,9], and PPARγ promotes fat storage and reduces plasma lipid levels, especially plasma levels of triglyceride [28,29]. We hypothesize that decrease in transcription and translation of *NCOA3* caused by polymorphism rs2076546 may lead to decrease in *PPARγ* expression level which may lead to increase in the plasma levels of triglyceride and hypertriglyceridemia. Hence, the association of rs2425955 with hypertriglyceridemia might be explained by the effect of rs2076548 which is in LD with rs2425955.

In addition, rs2425955 is located at the intron 1 of *NCOA3*. Hsia et al. found several functional E2F binding sites in the intron 1 and exon 1 of NCOA3 gene which were crucial for NCOA3 gene activation [35]. E2Fs, coactivated by NCOA3, is a transcription factor family that regulates genes expression in cell cycle progression and DNA synthesis [35–38]. Their data showed that these functional binding sites in the intron 1 may influence E2F activation, and enhanced activation of E2F could at least in part increase the expression of NCOA3 gene [35]. Previous studies reported that gene polymorphisms may regulate gene expression by changing the binding sites of transcription factors, and may further be associated with specific phenotypes [39,40]. We suppose that intron 1 SNPs of *NCOA3* may have potential effect on gene transcription. Based on the consideration, we intended to indentify putative E2F binding sites in intron 1 using the JASPAR database [41], and found that there was a putative binding site sequence (CGCGCCCCAGC) near the rs2425955. SNP rs2425955 was 36 bp away from the putative binding site. We suppose that polymorphism rs2425955 may influence steric conformation of chromosome region which contains the putative binding site or the two functional binding sites, and further have potential effect on transcription factor binding, which may influence gene transcription, expression and its biological function. However, functional studies are needed to clarify the potential role of intron 1 SNPs, especially rs2425955, on NCOA3 gene transcription and expression.

The reasons mentioned above may explain the association between rs2425955 and hypertriglyceridemia in our study. There is scarcely any information about the relationship between *NCOA3* SNPs and dyslipidemia in humans. Our sample size is relatively small, replication of the finding from this study with a larger sample is necessary to further explore the role of the target gene and its variation in the development of metabolic disorders.

Besides, SNP rs10485463 was associated with low-HDL cholesterolemia, and SNP rs6066394 was slightly associated with a lower risk of hypercholesterolemia in our study. However, in the analysis of inheritance models the association between the four SNPs and dyslipidemia (hypercholesterolemia and low-HDL cholesterolemia) was not observed after bonferroni correction. Therefore, whether the two SNPs (rs10485463 and rs6066394) are associated with dyslipidemia needs to be further studied.

## Conclusions

The four NCOA3 SNPs were associated with plasma levels of triglyceride, and except rs10485436, three other SNPs of NCOA3 gene were associated with a decreased risk of hypertriglyceridemia. In addition, SNP rs10485463 was associated with low-HDL cholesterolemia, and SNP rs6066394 was slightly associated with a lower risk of hypercholesterolemia. In conclusion, our present study revealed that the polymorphisms in *NCOA3* might be in association with dyslipidemia and serum lipid levels in the Chinese Han population.

## Materials and methods

### Study subjects

This study recruited participants from two sources, both in Jilin Province, Northeast of China: from the General Hospital of Jilin Chemical Group Corporation and from a survey of the prevalence and risk factors of chronic diseases among adults in Jilin. One hundred and ninety-two (192) Chinese Han subjects were randomly selected from individuals who received routine health examinations at the General Hospital of Jilin Chemical Group Corporation between September 2009 and June 2010. Three hundred and thirty- seven (337) subjects were recruited from a survey conducted in July 2012 on the prevalence and risk factors of chronic diseases among adults in Jilin Province. All study participants accepted general health examination included blood pressure, BMI, and plasma levels of lipids. A total of 529 (252 males and 277 females) subjects with an average age of 59 ± 10 years were included in this study. All subjects were unrelated Chinese Han.

Dyslipidemia was assessed according to the Guidelines on Prevention and Treatment of dyslipidemia in Chinese Adults [42]: hypertriglyceridemia: triglyceride (TG) ≥2.26 mmol/L;hypercholesterolemia: total cholesterol (TC) ≥6.22 mmol/L; Hyper-LDL cholesterolemia: LDL-C ≥4.14 mmol/L; Low-HDL cholesterolemia: HDL-C < 1.04 mmol/L.

The study was approved by the ethics committee of the School of Public Health, Jilin University, and informed consents were provided by all subjects.

### Tag SNP selection

NCOA3 gene is located in chromosomal region 20q12. Tag SNPs for *NCOA3* were chosen from the HapMap database (HapMap Genome Brower release # 24) [43]. In the public HapMap database (phase II Nov08, on NCBI B36 assembly, dbSNP b126) tag SNPs were selected under the following options: Han Chinese in Beijing population (CHB), r^2^ cut-off of 0.8, tagger pairwise as pairwise methods and MAF cut-off of 10 %. Four tag SNPs (rs2425955 G > T, rs6066394 T > C, rs10485463 C > G, and rs6094753 G > A) of *NCOA3* were chosen. The positions of rs2425955, rs6066394, rs10485463, and rs6094753 are intron 1, intron 1, intron 2, and intron 8 of the NCOA3 gene respectively.

### DNA extraction, purification, and SNP genotyping

Genomic DNA was extracted from peripheral blood samples using standard protocol (phenol/chloroform extraction), and the quality and quantity were determined by spectrophotometry.

Genotyping was conducted by polymerase chain reaction (PCR) and MALDI-TOF mass spectrometry [44] using the MassARRAY iPLEX System (Sequenom Inc., San Diego, CA).

### Data analysis

Clinical and biochemical data were expressed as mean ± SD or percentage. One sample Kolmogorov-Smirnov test was applied to evaluate whether TG, TC, LDL, and HDL were in normal distributions. Two variables, TG and HDL, were logarithmically transformed to conform to normality in order to allow covariance analysis. The association between the four *NCOA3* SNPs and quantitative clinical traits was assessed by covariance analysis. The goodness of fit Chi-square test was used to check if genotype distributions were in HWE. The Chi-square test was used to check if allele frequencies and genotype distributions were significantly different between dyslipidemia group and normal group. The associations between the allele of the four SNPs of *NCOA3* and dyslipidemia were evaluated by odds ratios and their 95 % confidence intervals (CI) using Chi-square test. Logistic regression analysis was used to detect associations between genotypes of *NCOA3* SNPs and dyslipidemia. When we explored the association using logistic regression without any adjustment, we set hypercholesterolemia, hypertriglyceridemia, low-HDL cholesterolemia, and hyper-LDL cholesterolemia as the dependent variables, and genotypes as independent variable. When we explored the association after adjustment for age and sex, we set hypercholesterolemia, hypertriglyceridemia, low-HDL cholesterolemia, and hyper-LDL cholesterolemia as the dependent variable, genotypes, sex, and age (continuous) as independent variable, and enter as method. The strength of any evident association was explored by calculating odds ratios together with their 95 % CI. Besides, the association between the four *NCOA3* SNPs and dyslipidemia in five inheritance models (codominant, dominant, recessive, overdominant, and additive model) was analyzed using SNPstats programs [45]. To reduce Type I error generated from the association analysis involving four SNPs in the five genetic models, bonferroni correction was applied to *P* values for correcting multiple testing (corrected *P*-value: 0.05/(4 SNPs × 5 different genetic models) = 0.0025). LD blocks of SNPs were constructed using SNP genotyping data in this study, and estimation of LD of 4 SNPs in *NCOA3* (D’ and r^2^ represent the degree of LD) was analyzed by haploview 4.2 software. The power of this study was evaluated by Power and Sample Size software version 3.1.2 [46, 47], and we found that our study had a 0.745 power to detect a significant difference between genotypes. All analyses were performed using SPSS 16.0 for Windows unless otherwise specified, and *P*-value <0.05 was considered statistically significant.

## Abbreviations

NCOA3: Nuclear receptor coactivator-3
SNP: Single nucleotide polymorphism
PPARγ: Peroxisome proliferator-activated receptor γ
VAT: Visceral adipose tissue
SCAT: Subcutaneous adipose tissue
HFD: High-fat diet
PGC-1α: PPARγ coactivator-1α
EE: Energy expenditure
TC: Total cholesterol
TG: Triglyceride
HDL-C: HDL cholesterol
LDL-C: LDL cholesterol
MAF: Minor allele frequency
OR: Odds ratio
CI: Confidence interval
SD: Standard deviation
LD: Linkage disequilibrium
C/EBP: CCAAT/enhancer-binding proteins
LPL: Endothelial lipoprotein lipase
FAT: Fatty acid translocase
UCP: Uncoupling protein
PCR: Polymerase chain reaction
HWE: Hardy-Weinberg equilibrium
BMI: Body mass index
